# Risk of first musculoskeletal disorder in Danish occupational fishermen – a register-based study

**DOI:** 10.1093/eurpub/ckac130.093

**Published:** 2022-10-25

**Authors:** LN Remmen, DH Christiansen, K Herttua, G Berg-Beckhoff

**Affiliations:** 1 Research Unit for Health Promotion, University of Southern Denmark, Esbjerg, Denmark; 2 Department of Occupational Medicine, Regional Hospital West Jutland, Herning, Denmark; 3 Department of Clinical Medicine, Aarhus University, Aarhus N, Denmark; 4 Center for Maritime Health and Society, University of Southern Demark, Esbejrg, Denmark

## Abstract

**Background:**

Occupational fishery increase risk of musculoskeletal disorders due to a combination of heavy workloads and strenuous settings. Scarce and inconsistent knowledge exists on work-related risk factors despite high prevalence is evident. The aim was to determine work-related risk factors for the first diagnosis of musculoskeletal disorders in Danish occupational fishermen.

**Methods:**

This study was a register-based cohort study. We extracted data the from Nationwide Danish registers on work affiliation and health data for all persons registered as occupational fishermen between 1994 and 2017. Job titles were retrieved from the Danish Occupational Cohort with eXposure (DOC*X). Time-to-event analysis using cox regression with age as timescale was applied.

**Results:**

Among 15.739 fishermen, forty percent (n = 6.218 cases) experienced first musculoskeletal disorder during 82.2 million person-years of follow-up. Adjusted gender-stratified analysis showed that male fishermen, who worked less than 5 years and more than 15 years had the highest significant risks of MSD (HR 2.40 (95%CI: 2.06, 2.80), HR: 2.40 (95%CI: 1.76, 2.35)) respectively, compared to working more than 20 years. In males, more years in workforce, a captain education and working part time significantly protected against first MSD, while shifting trades above three times increased risk. Women had estimates with greater uncertainties due to their small numbers in the industry.

**Conclusions:**

A high incidence of musculoskeletal disorders was found in Danish occupational fishermen between 1994-2017. Findings suggest a bimodal relationship between occupational fishermen seniority level and their risk of musculoskeletal disorder, where highest risk was seen at five years in trade, afterwards from lower estimate slowly increasing with accumulating years until highest occupational seniority, compared to more than twenty years in trade. Continued development actions of preventive measures are suggested.

**Key messages:**

- Persons who are working large proportions of their work-life within occupational fishery significantly increase the risk of experiencing first musculoskeletal disorder.
- High incidence of musculoskeletal disorder cases within Danish occupational fishery was shown, therefor further preventive actions towards work-related risk factors is necessary.

